# Supplementary material for: Risk factors and microbiological features of recurrent Escherichia coli bloodstream infections
Source: PLoS One. 2023 Jan 10;18(1):e0280196. doi: 10.1371/journal.pone.0280196 (PMC9831297; doi:10.1371/journal.pone.0280196)
Supplement: S1 Table — (DOCX) [file pone.0280196.s002.docx]

**Table S1.** Comparison of patients with early- and late-onset recurrent *Escherichia coli* BSIs

| Variables | Early recurrence (N = 29) | Late recurrence (N = 28) | *P* |
| --- | --- | --- | --- |
| Age, years | 74 (64–83) | 80 (71.5–84) | 0.318 |
| Sex, n (%) |  |  | 0.117 |
| Female | 16 (55.2) | 21 (75) |  |
| Male | 13 (44.8) | 7 (25) |  |
| Hospital acquired infection, n (%) | 12 (41.4) | 14 (50) | 0.514 |
| Transferred case, n (%) |  |  | 0.621 |
| Non-transferred | 17 (58.6) | 14 (50) |  |
| Long-term care facility | 3 (10.3) | 1 (3.6) |  |
| Acute care hospital | 5 (17.2) | 7 (25) |  |
| Clinic | 4 (13.8) | 6 (21.4) |  |
| Comorbidity, n (%) |  |  |  |
| Diabetes mellitus | 41 (27.5) | 9 (32.1) | 0.326 |
| Heart failure | 6 (4.0) | 2 (7.1) | 0.237 |
| Pulmonary disease | 3 (2.0) | 0 | >.999 |
| Chronic kidney disease | 15 (10.1) | 7 (25) | 0.025 |
| Liver cirrhosis | 3 (2.0) | 1 (3.57) | 0.194 |
| Cancer | 25 (16.8) | 8 (28.6) | 0.704 |
| Charlson comorbidity index | 2 (0-5) | 1.5 (0-3) | 0.878 |
| Primary focus of bacteremia, n (%) |  |  | 0.358 |
| Non-urinary tract infection | 10 (34.5) | 13 (46.4) |  |
| Urinary tract infection | 19 (65.5) | 15 (53.6) |  |
| Polymicrobial blood stream infection, n (%) | 1 (3.5) | 1 (3.6) | >.999 |
| Antibiotic use within 1 month before episode, n (%) | 26 (89.7) | 22 (78.6) | 0.297 |
| Appropriate empiciral antibiotic use, n (%) | 20 (69.0) | 20 (71.4) | 0.839 |
| Appropriate definite antibiotic use, n (%) | 24 (82.8) | 28 (100) | 0.052 |
| Duration of definite antibiotic use, days | 14 (12–19) | 14 (13–15) | 0.724 |
| Indwelling foley catheter in the previous 30 days, n (%) |  |  |  |
| Vascular catheter | 4 (13.8) | 2 (7.4) | 0.671 |
| Urinary catheter | 17 (58.6) | 16 (57.1) | 0.91 |
| *E. coli* with ESBL phenotype, n (%) | 15 (51.7) | 9 (32.1) | 0.134 |
| SOFA score | 6 (2–9) | 3 (2–6.5) | 0.234 |
| Time to recurrence since first episode | 6 (4–15.5) | 66.5 (44.3–134.3) | <0.001 |
| 30-day mortality, n (%) | 3 (10.3) | 0 | 0.237 |

BSI, bloodstream infection; ESBL, extended spectrum β-lactamase; SOFA, sequential organ failure assessment
