# Supplementary material for: Risk factors and microbiological features of recurrent Escherichia coli bloodstream infections
Source: PLoS One. 2023 Jan 10;18(1):e0280196. doi: 10.1371/journal.pone.0280196 (PMC9831297; doi:10.1371/journal.pone.0280196)
Supplement: S2 Table — (DOCX) [file pone.0280196.s003.docx]

**S2 Table.** **Whole genome sequencing data of ESBL–producing *E. coli* isolated from patients with BSI**

| No | Episode | ST | Resistance gene | Plasmid | Virulence gene |
| --- | --- | --- | --- | --- | --- |
| EC 0049 | Single | 131 | *aac(3)-IIa, catB3, aac(6')Ib-cr, bla*_OXA-1_*, bla*_CTX-M-15_ | Not detected | *nfaE, iha, gad, cnf1, iss, sat* |
| EC 0075 | Single | 131 | *bla*_CTX-M-27_ | IncFIA, IncFIB(AP001918), IncFIB(pLF82-PhagePlasmid), IncFII | *sat, iha, iss, gad* |
| EC 0131 | Single | 131 | *aacA4, catB3, aac(6')Ib-cr, bla*_CTX-M-15_*, bla*_OXA-1_ | Col(BS512), IncFIA, IncFIB(AP001918) | *iss, gad* |
| EC 0306 | Single | 131 | *aadA5, mph(A), sul1, dfrA17, catB3, bla*_CTX-M-15_*, bla*_OXA-1_*, aac(6')Ib-cr* | Col(BS512), Col156, IncFIA, IncFIB(AP001918) | *sat, senB, iha, iss, gad* |
| EC 0364 | Single | 131 | *aac(3)-IIa, aadA5, mph(A), sul1, dfrA17, tet(A), catB3, aac(6')Ib-cr, bla*_OXA-1_*, bla*_CTX-M-15_ | Col156, IncFIA, IncFIB(AP001918) | *cnf1, iha, gad, nfaE, iss, celb, senB, sat* |
| EC 0436 | Single | 131 | *aadA5, aac(3)-IIa, sul1, dfrA17, catB3, bla*_CTX-M-15_*, mph(A), bla*_OXA-1_*, aac(6')Ib-cr* | Col(BS512), IncFIA, IncFIB(AP001918) | *sat, iha, iss, gad* |
| EC 0500 | Single | 131 | *aadA5, aac(3)-IIa, sul1, dfrA17, tet(A), catB3, bla*_CTX-M-15_*, mph(A), bla*_OXA-1_*, aac(6')Ib-cr* | IncFIA, IncFIB(AP001918) | *cnf1, iha, gad, nfaE, iss, senB, sat* |
| EC 0530 | Single | 131 | *strA, strB, aac(3)-IIa, aadA5, mph(A), sul1, sul2, dfrA17, tet(A), catB3, bla*_CTX-M-15_*, bla*_OXA-1_*, aac(6')Ib-cr* | Col156, IncFIA, IncFIB(AP001918), IncFII(pRSB107) | *senB, astA, iss, gad* |
| EC 0745 | Single | 131 | *aac(3)-IIa, catB3, bla*_CTX-M-15_*, bla*_OXA-1_*, aac(6')Ib-cr* | IncFIA, IncFIB(AP001918) | *nfaE, iha, gad, cnf1, iss, sat* |
| EC 1064 | Single | 744 | *tet(A), tet(B), catA2, dfrA14, bla*_CTX-M-55_*, aac(3)-IIa* | IncFIB(AP001918), IncFIC(FII), IncX4 | *cma, gad* |
| EC 1346 | Single | 1193 | *strA, strB, aadA5, mph(A), sul1, sul2, dfrA17, tet(A), bla*_CTX-M-27_ | Col(BS512), Col156, IncFIA, IncFIB(AP001918) | *cnf1, ireA, iha, gad, vat, senB, sat* |
| EC 0295 | Early onset recurrent | 131 | *aac(3)-IId, blaTEM-1B, bla*_CTX-M-14_ | IncFIA, IncFIB(AP001918) | *sat, cnf1, iha, iss, gad* |
| EC 0355 | Early onset recurrent | 95 | *aadA5, aac(3)-IId, sul1, dfrA17, bla*_CTX-M-14_*, mph(A)* | Col156, IncB/O/K/Z, IncFIB(AP001918), IncFII(29) | *vat, ireA, gad, iss, senB* |
| EC 0511 | Early onset recurrent | 131 | *strA, strB, aadA5, mph(A), sul1, sul2, dfrA17, tet(A), bla*_CTX-M-27_ | Col(KPHS6), IncFIA, IncFIB(AP001918), IncFII(pRSB107) | *sat, iss, gad* |
| EC 0578 | Early onset recurrent | 131 | *aac(3)-IId, strA, strB, aadA5, mph(A), sul1, sul2, dfrA17, tet(A), bla*_CTX-M-14_*, bla*_TEM-1B_ | Col156, IncFIA, IncFIB(AP001918), IncFII(pRSB107) | *nfaE, iha, gad, cnf1, iss, senB, sat* |
| EC 0632 | Early onset recurrent | 131 | *bla*_CTX-M-27_ | Col156, IncFIA, IncFIB(AP001918), IncFII(pRSB107), IncI1-I(Gamma) | *senB, iss, gad* |
| EC 1132 | Early onset recurrent | 1193 | *mph(A), bla*^CTX-M-15^ | Col(BS512), IncFIA, IncFIB(AP001918), IncFII(pHN7A8) | *sat, vat, iha, senB, gad* |
| EC 1286 | Early onset recurrent | 131 | *aac(3)-IIa, aadA5, mph(A), sul1, dfrA17, tet(A), catB3, aac(6')Ib-cr, bla*_OXA-1_*, bla*_CTX-M-15_ | IncFIA, IncFIB(AP001918), IncI1-I(Gamma) | *nfaE, iha, gad, cnf1, iss, senB, sat* |
| EC 1317 | Early onset recurrent | 131 | *aac(3)-IIa, tet(A), catB3, aac(6')Ib-cr, bla*_OXA-1_*, bla*_CTX-M-15_ | IncB/O/K/Z, IncFIA, IncFIB(AP001918) | *sat, cnf1, iha, iss, gad* |
| EC 1398 | Early onset recurrent | 131 | *strA, strB, aac(3)-IId, aadA5, mph(A), sul1, sul2, dfrA17, tet(A), bla*_CTX-M-14_*, bla*_TEM-1B_ | Col156, IncFIA, IncFIB(AP001918), IncFII(pRSB107) | *nfaE, iha, cnf1, gad, iss, senB, sat* |
| EC 1508 | Early onset recurrent | 131 | *aadA5, aac(3)-IIa, mph(A), sul1, dfrA17, tet(A), catB3, bla*_CTX-M-15_*, bla*_OXA-1_*, aac(6')Ib-cr* | IncFIA, IncFIB(AP001918) | *cnf1, iha, gad, nfaE, iss, senB, sat* |
| EC 1631 | Early onset recurrent | 131 | *strA, strB, aadA5, mph(A), sul1, sul2, dfrA17, tet(A), bla*_CTX-M-27_ | IncFIA, IncFIB(AP001918), IncFII(pRSB107) | *senB, ireA, iha, iss, gad* |
| EC 0151 | Late onset recurrent | 131 | *aac(3)-IIa, catB3, aac(6')Ib-cr, bla*_OXA-1_*, bla*_CTX-M-15_*, tet(A)* | Col156, IncFIA, IncFIB(AP001918) | *cnf1, iha, nfaE, gad, iss, sat* |
| EC 1254 | Late onset recurrent | 648 | *aac(3)-IIa, aadA5, mph(A), sul1, dfrA17, tet(B), bla*_CTX-M-14_ | Col(MG828), IncFIA, IncFIB(AP001918), IncFII(pRSB107) | *eilA, lpfA, air* |
| EC 1256 | Late onset recurrent | 131 | *aac(3)-IIa, aadA5, mph(A), sul1, dfrA17, tet(A), catB3, aac(6')Ib-cr, bla*_OXA-1_*, bla*_CTX-M-15_ | Col156, ColpVC, IncFIA, IncFIB(AP001918) | *cnf1, iha, gad, iss, senB, sat* |
| EC 1448 | Late onset recurrent | 131 | *aac(3)-IId, strA, strB, aadA5, mph(A), sul1, sul2, dfrA17, tet(A), bla*_CTX-M-3_*, bla*_TEM-1B_ | IncFIB(AP001918), IncFII | *iha* |
| EC 1563 | Late onset recurrent | 131 | *aac(3)-IIa, aadA5, mph(A), sul1, dfrA17, tet(A), catB3, aac(6')Ib-cr, bla*_OXA-1_*, bla*_CTX-M-15_ | Col156, IncFIA, IncFIB(AP001918) | *cnf1, iha, gad, nfaE, iss, senB, sat* |

ESBL, extended spectrum β-lactamase; BSI, bloodstream infection; ST, sequence type
