# Supplementary material for: Risk factors and microbiological features of recurrent Escherichia coli bloodstream infections
Source: PLoS One. 2023 Jan 10;18(1):e0280196. doi: 10.1371/journal.pone.0280196 (PMC9831297; doi:10.1371/journal.pone.0280196)
Supplement: Attachment — Submitted filename: PONE-D-22-18648_reviewed.pdf [file pone.0280196.s004.pdf]

PLOS ONE

Risk factors and microbiological features of recurrent Escherichia coli bloodstream infections

--Manuscript Draft--

**Manuscript Number:**	PONE-D-22-18648	
**Article Type:**	Research Article	
**Full Title:**	Risk factors and microbiological features of recurrent Escherichia coli bloodstream infections	
**Short Title:**	Risk factors and microbiological features of recurrent Escherichia coli bloodstream infections	There is no shortening
**Corresponding Author:**	Yoon Soo Park, M.D., Ph. D.<br>NHIS Ilsan Hospital<br>Goyang-si, Gyeonggi-do KOREA, REPUBLIC OF	
**Keywords:**	recurrent bloodstream infection; Escherichia coli; risk factors, whole genome sequencing	
**Abstract:**	Background : Understanding the risk factors and microbiological features in recurrent Escherichia coli BSI is helpful for clinicians.<br>Methods : Data of patients with E. coil BSI from 2017 to 2018 were collected. Antimicrobial resistance rates of E. coli were determined. We also identify the ST131 and ESBL genotype to evaluate the molecular epidemiology of E. coli . Whole genome sequencing was conducted on the available ESBL–producing E. coli samples.<br>Findings : Of 808 patients with E. coli BSI, 57 (6.31%) experienced recurrence; 29 developed at 4–30 days after initial BSI (early onset recurrence) and 28 at 31–270 days after initial BSI (late onset recurrence). One hundred forty-nine patients with single episode, whose samples were available for determining the molecular epidemiology, were selected for comparison. Vascular catheterization (adjusted odds ratio [aOR], 4.588; 95% confidence interval [CI], 1.049–20.068), ESBL phenotype (aOR, 2.037; 95% CI, 1.037–3.999) and SOFA score ≥9 (aOR, 3.210; 95% CI, 1.359–7.581) were independent risk factors for recurrence. The proportion of ST131 and ESBL genotype was highest in early onset recurrent BSI (41.4% and 41.4%, respectively), from which E. coil had the highest resistance rates to most antimicrobial agents. Whole genome sequencing on 27 of ESBL–producing E. coli (11 from single episode, 11 from early onset recurrence, and 5 from late onset recurrence) demonstrated that various virulence factors, resistant genes, and plasmid types existed in isolates from all types of BSI.<br>Conclusion : Risk factors contributing to the recurrence and microbiological features of E. coli causing recurrent BSI may be helpful for management planning in the clinical setting.	Itaics<br>grammaical error
**Order of Authors:**	Yong Chan Kim	
	Heun Choi	
	Young Ah Kim	
	Yoon Soo Park, M.D., Ph. D.	
	Young Hee Seo	
	Hyukmin Lee	
	Kyungwon Lee	
**Additional Information:**		
**Question**	**Response**	
**Financial Disclosure**<br><br>Enter a financial disclosure statement that	This work was supported by the National Health Insurance Service Ilsan Hospital grant (No. NHIMC2019CR012 to YAK). The funder played no role in the study design, data analysis, or preparation of the manuscript.	

describes the sources of funding for the work included in this submission. Review the submission guidelines for detailed requirements. View published research articles from *PLOS ONE* for specific examples.<br><br>This statement is required for submission and **will appear in the published article** if the submission is accepted. Please make sure it is accurate.<br><br>**Unfunded studies**<br>Enter: *The author(s) received no specific funding for this work.*<br><br>**Funded studies**<br>Enter a statement with the following details:<br>• Initials of the authors who received each award<br>• Grant numbers awarded to each author<br>• The full name of each funder<br>• URL of each funder website<br>• Did the sponsors or funders play any role in the study design, data collection and analysis, decision to publish, or preparation of the manuscript?<br>• **NO** - Include this sentence at the end of your statement: *The funders had no role in study design, data collection and analysis, decision to publish, or preparation of the manuscript.*<br>• **YES** - Specify the role(s) played.<br><br>* typeset	
**Competing Interests**<br><br>Use the instructions below to enter a competing interest statement for this submission. On behalf of all authors, disclose any competing interests that could be perceived to bias this work—acknowledging all financial support and any other relevant financial or non-financial competing interests.<br><br>This statement is required for submission and **will appear in the published article** if the submission is accepted. Please make sure it is accurate and that any funding	The authors have declared that no competing interests exist.

sources listed in your Funding Information later in the submission form are also declared in your Financial Disclosure statement.<br><br>View published research articles from *PLOS ONE* for specific examples.<br><br>**NO authors have competing interests**<br><br>Enter: *The authors have declared that no competing interests exist.*<br><br>**Authors with competing interests**<br><br>Enter competing interest details beginning with this statement:<br><br>*I have read the journal's policy and the authors of this manuscript have the following competing interests: [insert competing interests here]*<br><br>* typeset	
**Ethics Statement**<br><br>Enter an ethics statement for this submission. This statement is required if the study involved:<br><br>• Human participants<br>• Human specimens or tissue<br>• Vertebrate animals or cephalopods<br>• Vertebrate embryos or tissues<br>• Field research<br><br>Write "N/A" if the submission does not require an ethics statement.<br><br>General guidance is provided below. Consult the submission guidelines for detailed instructions. **Make sure that all information entered here is included in the Methods section of the manuscript.**	The study protocol was approved by the Institutional Review Board (NHIMC-2019-01-018) of the National Health Insurance Service at Ilsan Hospital. As this study was retrospective and data were anonymous, the Institutional Review Board waived the requirement for written informed consent from the participants.

**Format for specific study types**<br><br>**Human Subject Research (involving human participants and/or tissue)**<br>• Give the name of the institutional review board or ethics committee that approved the study<br>• Include the approval number and/or a statement indicating approval of this research<br>• Indicate the form of consent obtained (written/oral) or the reason that consent was not obtained (e.g. the data were analyzed anonymously)<br><br>**Animal Research (involving vertebrate animals, embryos or tissues)**<br>• Provide the name of the Institutional Animal Care and Use Committee (IACUC) or other relevant ethics board that reviewed the study protocol, and indicate whether they approved this research or granted a formal waiver of ethical approval<br>• Include an approval number if one was obtained<br>• If the study involved *non-human primates*, add *additional details* about animal welfare and steps taken to ameliorate suffering<br>• If anesthesia, euthanasia, or any kind of animal sacrifice is part of the study, include briefly which substances and/or methods were applied<br><br>**Field Research**<br><br>Include the following details if this study involves the collection of plant, animal, or other materials from a natural setting:<br>• Field permit number<br>• Name of the institution or relevant body that granted permission	
**Data Availability**<br><br>Authors are required to make all data underlying the findings described fully available, without restriction, and from the time of publication. PLOS allows rare exceptions to address legal and ethical concerns. See the PLOS Data Policy and FAQ for detailed information.	Yes - all data are fully available without restriction

A Data Availability Statement describing where the data can be found is required at submission. Your answers to this question constitute the Data Availability Statement and **will be published in the article**, if accepted.<br><br>**Important:** Stating 'data available on request from the author' is not sufficient. If your data are only available upon request, select 'No' for the first question and explain your exceptional situation in the text box.<br><br>Do the authors confirm that all data underlying the findings described in their manuscript are fully available without restriction?	
**Describe where the data may be found in full sentences. If you are copying our sample text, replace any instances of XXX with the appropriate details.**<br><br>• If the data are **held or will be held in a public repository**, include URLs, accession numbers or DOIs. If this information will only be available after acceptance, indicate this by ticking the box below. For example: *All XXX files are available from the XXX database (accession number(s) XXX, XXX.*).<br>• If the data are all contained **within the manuscript and/or Supporting Information files**, enter the following: *All relevant data are within the manuscript and its Supporting Information files.*<br>• If neither of these applies but you are able to provide **details of access elsewhere**, with or without limitations, please do so. For example:<br><br>*Data cannot be shared publicly because of [XXX]. Data are available from the XXX Institutional Data Access / Ethics Committee (contact via XXX) for researchers who meet the criteria for access to confidential data.*<br><br>*The data underlying the results presented in the study are available from (include the name of the third party*	All data generated or analyzed during this study are included in this published article (and its supplementary files).

and contact information or URL).<br>• This text is appropriate if the data are owned by a third party and authors do not have permission to share the data.<br><br>* typeset	
Additional data availability information:	

Manuscript
Click here to access/download;Manuscript;renamed_14cd7.docx

Risk factors and microbiological features of recurrent *Escherichia coli* bloodstream infections

Yong Chan Kim, M.D.[1†], Heun Choi, M.D.[2†], Young Ah Kim, M.D., Ph.D.[3*], Yoon Soo Park, M.D., Ph.D.[1*], Young Hee Seo, B.D.[4], Hyukmin Lee, M.D., Ph.D.[4], and Kyungwon Lee M. D., Ph.D.[4,5]

[1]Department of Internal Medicine, Division of Infectious disease, Yongin Severance Hospital, Yonsei University College of Medicine
[2]Department of Infectious Diseases, National Health Insurance Service Ilsan Hospital
[3]Department of Laboratory Medicine, National Health Insurance Service Ilsan Hospital
[4]Department of Laboratory Medicine and Research Institute of Bacterial Resistance, Yonsei University College of Medicine, Seoul, Republic of Korea
[5]Seoul Clinical Laboratories Academy

[*]Correspondence to Young Ah Kim or Yoon Soo Park
Young Ah Kim, M.D., Ph.D.
Department of Laboratory Medicine, National Health Insurance Service Ilsan Hospital, Goyang-si, 10444, Republic of Korea
Tel: +82-10-6635-3581
Fax: +82-31-900-0049
E-mail: YAKIM@nhimc.or.kr
or
Yoon Soo Park, M.D., Ph.D.

Department of Internal Medicine, Division of Infectious disease, Yongin Severance Hospital, Yonsei University College of Medicine, Yongin-si, 16995, Republic of Korea

Tel: +82-10-8502-5828

Fax: +82-31-5189-8567

E-mail: YSPARKOK2@yuhs.ac

†Yong Chan Kim and Heun Choi contributed equally to this manuscript.

Abstract

Background**:** Understanding the risk factors and microbiological features in recurrent *Escherichia coli* BSI is helpful for clinicians.

Methods**:** Data of patients with *E. coil* BSI from 2017 to 2018 were collected. Antimicrobial resistance rates of *E. coli* were determined. We also identify the ST131 and ESBL genotype to evaluate the molecular epidemiology of *E. coli*. Whole genome sequencing was conducted on the available ESBL–producing *E. coli* samples.

Findings**:** Of 808 patients with *E. coli* BSI, 57 (6.31%) experienced recurrence; 29 developed at 4–30 days after initial BSI (early onset recurrence) and 28 at 31–270 days after initial BSI (late onset recurrence). One hundred forty-nine patients with single episode, whose samples were available for determining the molecular epidemiology, were selected for comparison. Vascular catheterization (adjusted odds ratio [aOR], 4.588; 95% confidence interval [CI], 1.049–20.068), ESBL phenotype (aOR, 2.037; 95% CI, 1.037–3.999) and SOFA score ≥9 (aOR, 3.210; 95% CI, 1.359–7.581) were independent risk factors for recurrence. The proportion of ST131 and ESBL genotype was highest in early onset recurrent BSI (41.4% and 41.4%, respectively), from which *E. coil* had the highest resistance rates to most antimicrobial agents. Whole genome sequencing on 27 of ESBL–producing *E. coli* (11 from single episode, 11 from early onset recurrence, and 5 from late onset recurrence) demonstrated that various virulence factors, resistant genes, and plasmid types existed in isolates from all types of BSI.

Conclusion**:** Risk factors contributing to the recurrence and microbiological features of *E. coli* causing recurrent BSI may be helpful for management planning in the clinical setting.

Keywords: recurrent bloodstream infection; *Escherichia coli*; risk factors, whole genome sequencing

Introduction

Escherichia coli is a common gram-negative bacteria that is component of the normal gut microbiota, but this pathogen may also cause blood stream infections (BSI) in humans [1]. *E. coli* has been identified as the leading causative agent in both community and hospital acquired BSI for the last decade [2-4]. The proportion of BSI episodes caused by *E. coli* reached as high as 27.1% over this period with an incidence rate of more than 48.0 per 100,000 people per year in high-income countries [5]. A study in England suggests that BSI caused by *E. coli* is increasing, and reported that there was a 6% increase in incidence between 2012/13 and 2013/14[2].

E. coli is one of the most common pathogens isolated from recurrent BSI[6-8], with the rates of recurrence in these infections ranging from 3.1%[9] to 9.9%[10]. Several factors have been shown to be associated with *E. coli*-based BSI recurrence [6,7,9-11], including hematological malignancy, non-urinary tract infection, inadequate antibiotic therapy, and the presence of a vascular catheter. Antibiotic resistance has also been shown to be associated with an increase in recurrent BSI [12]. In particular, the presence of multidrug-resistant extended-spectrum β-lactamase (ESBL)-producing *E. coli* often prevent effective empirical antibiotic therapy in these patients and restrict definite antibiotic therapy for recurrent BSI [13]. In addition, the presence of several virulence factors has been linked to recurrence, as they prevent host-immune reactions resulting in delayed or failed clearance of these pathogens [9].

Recurrence is a significant problem for *E. coli* BSI, which may require re-admission to the hospital thereby adding to the healthcare costs of this condition and potentially increasing the mortality rate of these infections. Therefore, it is important to investigate the prevalence, clinical characteristics, and microbiological features of recurrent BSI caused by *E. coli*. This

may be helpful in the planning management and clinical follow-up for these types of infections. However, recent information for the recurrence, especially after the widespread dissemination of ESBL–producing *E. coli*, is limited.

The aim of this study was to describe the current clinical and microbiological characteristics of patients with recurrent *E. coli* BSI in South Korea, where the *E. coli* strains isolated from blood specimens exhibit high rates of resistance to several antimicrobial agents.

Material and methods

Study design and population

We completed a retrospective study of recurrent and single episode *E. coli* BSIs using data collected by the National Health Insurance Service at Ilsan Hospital in South Korea between January 2017 and December 2018. Our study cohort consisted of adult patients (age ≥18 years) who presented at the Ilsan Hospital with positive blood cultures for *E. coli* at least once over the course of a two-year period. These patients were followed up over the 270 days following their initial *E. coli* BSI episode. Patients who died or were transferred to another hospital within three days of their initial diagnosis were excluded, as were patients who were expected to have recurrent bacteremia due to specific infection, such as infective endocarditis, mycotic aneurysm, or endovascular graft infection. All patients were only included once for each BSI episode and the demographic and clinical characteristics for each participant were extracted from their electronic medical records.

In a retrospective study how were the patient followed up?

Definition and classification of recurrent BSI

Recurrent BSI was defined as a positive blood culture from a specimen collected 4 days or later after the first infection. Recurrence was subdivided into early onset (4–30 days after

initial BSI) and late onset (31–270 days after initial BSI) [14]. Infections were also classified as community- or hospital-acquired with community-acquired BSI being defined as any infection occurring within 48 h of admission. Infections that developed more than 48 h after admission were defined as hospital-acquired BSI. Comorbidities were defined using the International Classification of Diseases, 10th revision and the Charlson comorbidity index, which predicts the 10-year mortality in patients who may have a range of comorbid conditions [15], was used to quantify a patient's burden of disease. Prior antibiotic use was defined as use of any antimicrobials for ≥ 3 days in the previous 30 days, and the primary focus of each case of BSI was classified according to the Centers for Disease Control and Prevention/National Healthcare Safety Network surveillance criteria [16]. Empirical antibiotics were regarded to be appropriate if administered within one calendar day of culture and the pathogen was susceptible to any of antibiotics used. Appropriate definite antibiotic therapy was defined as the administration of antibiotics based on the susceptibility, within one calendar day of the susceptibility results becoming available. We classified recurrence as a relapse when a strain from the second episode had an identical PFGE pattern to that of the initial episode. Recurrence was considered a reinfection when the PFGE pattern was different between the initial and subsequent episodes.

Microbiological analysis

Blood cultures was performed using BacT/ALERT FA/NA (BioMerieux Inc., Durham, NC, USA) with the blood bottles being incubated at 37 °C for five days. Individual species were identified using a MALDI Biotyper MALDI-TOF MS (Bruker Daltonik, Bremen, Germany), and antimicrobial susceptibility was evaluated using a MicroScan WalkAway plus system (Beckman Coulter, Inc., West Sacramento, CA, USA) and MicroScan Neg Breakpoint

Combo Type 44 panel (Siemens Healthcare Diagnostics, Inc., West Sacramento, CA, USA). The susceptibility results were interpreted using the guidelines set out by the Clinical and Laboratory Standards Institute [17].

We performed polymerase chain reaction (PCR)-sequencing of O25b and O16 to determine the sequence type (ST) 131, and a DNA region corresponding to CTX-M, SHV, and TEM was sequenced to determine ESBL genotype. PFGE was performed on eight pairs of late onset recurrence samples and used to distinguish between relapse and reinfection in these patients. The PFGE patterns were analyzed using InfoQuest FP software (Bio-Rad) and used to generate a dendrogram based on the unweighted pair group method, with an arithmetic average (UPGMA) from the Dice coefficient with 1% band position tolerance and 0.5% optimization settings [18].

We evaluated the ST, virulence factor, plasmid type, and antimicrobial resistance genes in each of the ESBL–producing *E. coli* using whole genome sequencing (WGS). WGS was performed on ESBL–producing *E. coli* isolated from patients with recurrent BSI and single episode BSI as follows. The DNA of freshly subcultured isolates was extracted using GenElute™ Bacterial Genomic DNA Kit (Sigma-Aldrich, St. Louis, MO), and 8 μg of input genomic DNA was then used to produce the relevant sequencing libraries. These whole genome libraries were then sequenced using a NextSeq 550 instrument (Illumina, San Diego, CA, USA), and sequences were assembled using Spades (version 3.11.1) and annotated with Prokka (version 1.13.7). The data from the multilocus sequence typing (MLST), plasmid typing, virulence, and resistance gene evaluations were obtained from the center for genomic epidemiology website (www.genomicepidemiology.org) The virulence score was calculated as the sum of all virulence factors, as described by Johnson et al [19].

Statistical analysis

The results of the categorical data are presented as numbers with percentages whereas those of the continuous data are reported as the median and IQR. The continuous variables were compared using the Mann–Whitney U test and the categorical variables were compared using the chi-squared test. Risk factors for recurrent BSI were identified by logistic regression analysis, and significant variables from the univariate analysis were then included in a multivariate logistic regression analysis. Results were considered statistically significant at $P < 0.05$. All statistical analyses were performed using SPSS 23.0 software (SPSS, Chicago, IL, USA).

Ethics statement

The study protocol was approved by the Institutional Review Board (NHIMC-2019-01-018) of the National Health Insurance Service at Ilsan Hospital. As this study was retrospective and data were anonymous, the Institutional Review Board waived the requirement for written informed consent from the participants.

Results

Clinical characteristics of patients with recurrent and single episode E. coli BSI

We identified a total of 808 patients with *E. coli* BSI admitted in the National Health Insurance Service Ilsan Hospital over the evaluation period, with 57 (6.31%) of these patients experiencing at least one recurrent episode. We then narrowed our evaluation cohort to the clinical characteristics of 206 patients, including 57 patients with recurrent BSI and 149 single episode patients whose samples were available for ST 131 sequencing and ESBL genotyping (Table 1). The median age of this cohort was 77 (interquartile range [IQR], 71–

84) and 78 (IQR, 67–85) in the recurrent episode and single episode groups, respectively. We did note that there was a slight female sex bias (64.9% in recurrent episode group and 71.1% in single episode group) and that the proportion of liver cirrhosis was higher in the recurrent episode group (N = 6, 10.5%) than in the single episode group (N = 3, 2.0%). The Charlson comorbidity index was also shown to be higher in patients with recurrent BSI (2, IQR, 0–4) when compared to those with single episode BSI (1, IQR, 0–2). Patients who had a vascular catheter were more likely to experience recurrence (N = 6, 10.5% vs N = 3, 2.0%) and *E. coli* with the ESBL phenotype were more frequently isolated from the recurrent samples (N = 24, 42.1% vs N = 37, 24.8%). The sequential organ failure assessment (SOFA) score was also higher in recurrent episode group (4, IQR, 2–8) than in the single episode group (3, IQR, 1–6), but mortality rates did not differ significantly between these groups ($P > 0.999$).

The 57 recurrent *E. coli* BSI cases were subdivided into early onset recurrence (29) and late onset recurrence (28). The median time to recurrence following the first episode was 6 days (IQR, 4–15.5) and 66.5 days (IQR, 44.3–134.3) in the early onset and late onset recurrence groups, respectively. Supplementary Table 1 describes the direct comparison of these two groups.

Table 1. Comparison of patients with recurrent *Escherichia coli* BSI and single episode *E. coli* BSI

Variables	Total (N = 206)	Single episode (N = 149)	Recurrent episode (N = 57)	*P*
Age, years	78 (70–84)	78 (67–85)	77 (71–84)	0.861
Sex, n (%)				0.385
Female	143 (69.42)	106 (71.1)	37 (64.9)	
Male	63 (30.6)	43 (28.9)	20 (35.1)	

Hospital acquired infection, n (%)	90 (43.7)	64 (43.0)	26 (45.6)	0.731
Transferred case, n (%)				0.894
Non-transferred	114 (55.3)	83 (55.7)	31 (54.4)	
Long-term care facility	17 (8.3)	13 (8.7)	4 (7.0)	
Acute care hospital	37 (18.0)	25 (16.8)	12 (21.1)	
Clinic	38 (18.5)	28 (18.8)	10 (17.5)	
Comorbidity, n (%)				
Diabetes mellitus	56 (27.2)	41 (27.5)	15 (26.3)	0.862
Heart failure	8 (3.9)	6 (4.0)	2 (3.5)	>.999
Pulmonary disease	4 (1.9)	3 (2.0)	1 (1.8)	>.999
Chronic kidney disease	23 (11.2)	15 (10.1)	8 (14.0)	0.419
Liver cirrhosis	9 (4.4)	3 (2.0)	6 (10.5)	0.015
Malignancy	40 (19.4)	25 (16.8)	15 (26.3)	0.122
Charlson comorbidity index	1 (0–3)	1 (0–2)	2 (0–4)	0.013
Primary focus of bacteremia, n (%)				0.114
Non-urinary tract infection	66 (32.0)	43 (28.9)	23 (40.4)	
Urinary tract infection	140 (68.0)	106 (71.1)	34 (59.7)	
Polymicrobial blood stream infection, n (%)	11 (5.3)	9 (6.0)	2 (3.5)	0.731
Prior antibiotic use, n (%)	180 (87.4)	132 (88.6)	48 (84.2)	0.397
Appropriate empirical antibiotic use, n (%)	152 (73.8)	112 (75.2)	40 (70.2)	0.466
Appropriate definite antibiotic use, n (%)	183 (88.8)	131 (87.9)	52 (91.2)	0.5
Duration of definite antibiotic use, days	14 (12–16)	14 (11–15)	14 (13–17)	0.126
Indwelling catheter in the previous 30 days, n (%)				
Vascular catheter	9 (4.4)	3 (2.0)	6 (10.7)	0.014
Urinary catheter	128 (62.1)	95 (63.8)	33 (57.9)	0.438
E. coli with ESBL phenotype, n (%)	61 (29.6)	37 (24.8)	24 (42.1)	0.015
SOFA score	3 (1–7)	3 (1–6)	4 (2–8)	0.035
30-day mortality, n (%)	13 (6.3)	10 (6.7)	3 (5.3)	>.999

BSI, bloodstream infection; ESBL, extended spectrum β-lactamase; SOFA, sequential organ failure assessment

Risk factors for recurrent E. coli BSI

Univariate logistic regression analysis demonstrated that liver cirrhosis, Charlson comorbidity index ≥3, vascular catheter, ESBL phenotype, and SOFA score ≥9 were all

associated with recurrent *E. coli* BSI (Table 2). The multivariate analysis identified the vascular catheter (adjusted odds ratio [aOR], 4.588; 95% confidence interval [CI], 1.049–20.068), ESBL phenotype (aOR, 2.037; 95% CI, 1.037–3.999), and SOFA score ≥9 (aOR, 3.210; 95% CI, 1.359–7.581) as independent risk factors for recurrent BSI.

Table 2. Risk factors for recurrent *Escherichia coli* BSI

Variables	Univariate analysis		Multivariate analysis	
	OR (95% CI)	*P*	Adjusted OR (95% CI)	*P*
Age≥ 65	1.577 (0.703–3.538)	0.269	1.579 (0.663–3.763)	0.302
Sex				
Female	Reference		Reference	
Male	1.332 (0.696–2.550)	0.386	1.098 (0.533–2.265)	0.799
Hospital acquired infection	1.114 (0.603–2.058)	0.731		
Transferred case				
Non-transferred	Reference			
Long-term care facility	0.824 (0.25–2.719)	0.751		
Acute care hospital	1.285 (0.576–2.867)	0.54		
Clinic	0.956 (0.416–2.196)	0.916		
Comorbidity				
Diabetes mellitus	0.941 (0.472–1.877)	0.862		
Heart failure	0.867 (0.17–4.424)	0.863		
Pulmonary disease	0.869 (0.089–8.531)	0.904		
Chronic kidney disease	1.459 (0.582–3.654)	0.42		
Liver cirrhosis	5.725 (1.381–23.735)	0.016		
Cancer	1.772 (0.854–3.675)	0.124		
Charlson comorbidity index≥ 3	1.973 (1.028–3.788)	0.041	1.591 (0.791–3.199)	0.193
Primary focus of bacteremia				
Non-urinary tract infection	Reference			
Urinary tract infection	0.6 (0.317–1.134)	0.116		
Polymicrobial bloodstream infection	0.566 (0.118–2.702)	0.475		
Antibiotic use ≤1 month before episode	1.456 (0.608–3.485)	0.399		
Appropriate empirical antibiotic use	0.777 (0.394–1.532)	0.467		
Appropriate definite antibiotic use	1.429 (0.504–4.049)	0.502		

Duration of definite antibiotic use	1.021 (0.995–1.047)	0.109		
Indwelling catheter in 30 days				
Vascular catheter	5.84 (1.408–24.222)	0.015	4.588 (1.049–20.068)	0.043
Urinary catheter	0.782 (0.419–1.457)	0.438		
Tracheal tube	0.867 (0.17–4.424)	0.863		
Nasogastric tube	1.456 (0.608–3.485)	0.399		
E. coli with ESBL phenotype	2.201 (1.156–4.191)	0.016	2.037 (1.037–3.999)	0.039
SOFA score ≥9	3.406 (1.487–7.804)	0.004	3.210 (1.359–7.581)	0.008

BSI, bloodstream infection; OR, odd ratio; CI, confidence interval, ESBL, extended spectrum β-lactamase; SOFA, sequential organ failure assessment

Molecular epidemiology and antimicrobial resistance of E. coli strains

Evaluation of the 206 isolates tested revealed that 53 (25.7%) could be classified as ST131, comprising ST131–025 (N = 47) and ST131–016 (N = 6) (Table 3). ST131 strains were more frequently observed in the early onset group (N = 12, 41.4%) when compared to the late onset recurrent episode (N = 7, 25.0%) and single episode (N = 34, 22.8%) groups. *E. coli* with an ESBL genotype were also more commonly observed in the early onset recurrent episode (N = 12, 41.4%) group when compared with either the late onset recurrent episode (N = 7, 25%) or single episode BSI (N = 25, 16.8%) groups. CTX-M-15 (N = 25, 56.8%) was the most common ESBL genotype, followed by CTX-M-14 (N = 11, 25%).

E. coli isolated from the early onset recurrent BSI group had higher resistance rates to most antimicrobial agents than those isolated from either the late onset recurrent BSI or single episode BSI groups (Figure 1). Resistance rates to piperacillin/tazobactam, cefoxitin, minocycline, chloramphenicol, fosfomycin, and tigecycline were all very low in all three groups. There were no amikacin-, colistin-, or carbapenem-resistant strains detected in this study.

Table 3. The molecular epidemiology of the *Escherichia coli* specimens isolated from patients with BSI

What statistical test was applied to note significant differences ?

	Single episode blood stream infection (N = 149)	Early onset recurrent blood stream infection (N = 29)	Late onset recurrent blood stream infection (N = 28)
ST131, n (%)	34 (22.8)	12 (41.4)	7 (25)
O25	29 (19.5)	12 (41.4)	6 (21.4)
O16	5 (3.4)	0	1 (3.6)
ESBL genotype, n (%)	25 (16.8)	12 (41.4)	7 (25)
CTX-M-3	0	0	1 (3.6)
CTX-M-14	5 (3.4)	3 (10.3)	3 (10.7)
CTX-M-15	17 (11.4)	5 (17.2)	3 (10.7)
CTX-M-27	2 (1.3)	4 (13.8)	0
CTX-M-55	1 (0.7)	0	0

BSI, bloodstream infection; ST, sequence type; ESBL, extended spectrum β-lactamase

Figure 1. Antimicrobial resistance rates of the *Escherichia coli* specimens isolated from patients with bloodstream infections (N = 206). Abbreviations: BSI, bloodstream infection; Amp, ampicillin; Sam, ampicillin/sulbactam; Pip, piperacillin; Tzp, piperacillin/tazobactam; Amc, amoxicillin/clavulanic acid; Cxm, cefuroxime; Ctx, cefotaxime; Caz, ceftazidime; Fep, cefepime; Fox, cefoxitin; Atm, aztreonam; Dor, doripenem; Mem, meropenem; Imp, imipenem; Lev, levofloxacin; Cip, ciproflroxacin; Gen, gentamicin; Tob, tobramycin; Amk, amikacin; Mno, minocycline; Tet, tetracycline; Sxt, cotrimoxazole; Chlor, chloramphenicol; Fos, fosfomycin; Tgc, tigecycline; Col, colistin; ESBL, extended spectrum β-lactamase.

WGS of ESBL–producing E. coli

Our cohort included a total of 27 ESBL–producing *E. coli* strains suitable for whole genome sequencing (WGS); 11 from single episode samples, 11 from early onset recurrent episode samples, and 5 from late onset recurrent episode samples. Various antimicrobial resistance genes, plasmid types, and virulence factors were detected in each of the isolates from each of the different BSI groups (Table 4). These included β-lactam (*bla*), streptomycin (*str*), aminoglycoside (*aac*, *aad*), macrolide (*mph*), quinolone (*aac(6')Ib-cr*), trimethoprim-sulfamethoxazole (*dfr*, *sul*), tetracycline (*tet*), and chloramphenicol (*cat*) resistance genes. The IncFIA, IncFIB, and IncFII were the most common plasmid type in these samples, but there was no dominant plasmid type. We also detected a wide range of virulence genes, with *gad* being the most common, followed by *iss*, *iha*, and *sat*. The median virulence scores were 5 (IQR, 3–7), 4.5 (IQR, 1–6), and 5 (IQR, 2–8) for early onset recurrent, late onset recurrent, and single episode BSI, respectively.

Table 4. Whole genome sequencing data of ESBL–producing *E. coli* isolated from patients with BSI Too crowded table. A compiled common groups may be considered

No	Episode	ST	Resistance gene	Plasmid	Virulence gene
EC 0049	Single	131	*aac(3)-IIa, catB3, aac(6')Ib-cr, bla*$_{\text{OXA-1}}$, *bla*$_{\text{CTX-M-15}}$	Not detected	*nfaE, iha, gad, cnf1, iss, sat*
EC 0075	Single	131	*bla*$_{\text{CTX-M-27}}$	IncFIA, IncFIB(AP001918), IncFIB(pLF82-PhagePlasmid), IncFII	*sat, iha, iss, gad*
EC 0131	Single	131	*aacA4, catB3, aac(6')Ib-cr, bla*$_{\text{CTX-M-15}}$, *bla*$_{\text{OXA-1}}$	Col(BS512), IncFIA, IncFIB(AP001918)	*iss, gad*
EC 0306	Single	131	*aadA5, mph(A), sul1, dfrA17, catB3, bla*$_{\text{CTX-M-15}}$, *bla*$_{\text{OXA-1}}$, *aac(6')Ib-cr*	Col(BS512), Col156, IncFIA, IncFIB(AP001918)	*sat, senB, iha, iss, gad*
EC 0364	Single	131	*aac(3)-IIa, aadA5, mph(A), sul1, dfrA17, tet(A), catB3, aac(6')Ib-cr, bla*$_{\text{OXA-1}}$, *bla*$_{\text{CTX-M-15}}$	Col156, IncFIA, IncFIB(AP001918)	*cnf1, iha, gad, nfaE, iss, celb, senB, sat*
EC 0436	Single	131	*aadA5, aac(3)-IIa, sul1, dfrA17, catB3, bla*$_{\text{CTX-M-15}}$, *mph(A), bla*$_{\text{OXA-1}}$, *aac(6')Ib-cr*	Col(BS512), IncFIA, IncFIB(AP001918)	*sat, iha, iss, gad*
EC 0500	Single	131	*aadA5, aac(3)-IIa, sul1, dfrA17, tet(A), catB3, bla*$_{\text{CTX-M-15}}$, *mph(A), bla*$_{\text{OXA-1}}$, *aac(6')Ib-cr*	IncFIA, IncFIB(AP001918)	*cnf1, iha, gad, nfaE, iss, senB, sat*
EC 0530	Single	131	*strA, strB, aac(3)-IIa, aadA5, mph(A), sul1, sul2, dfrA17, tet(A), catB3, bla*$_{\text{CTX-M-15}}$, *bla*$_{\text{OXA-1}}$, *aac(6')Ib-cr*	Col156, IncFIA, IncFIB(AP001918), IncFII(pRSB107)	*senB, astA, iss, gad*
EC 0745	Single	131	*aac(3)-IIa, catB3, bla*$_{\text{CTX-M-15}}$, *bla*$_{\text{OXA-1}}$, *aac(6')Ib-cr*	IncFIA, IncFIB(AP001918)	*nfaE, iha, gad, cnf1, iss, sat*
EC 1064	Single	744	*tet(A), tet(B), catA2, dfrA14, bla*$_{\text{CTX-M-55}}$, *aac(3)-IIa*	IncFIB(AP001918), IncFIC(FII), IncX4	*cma, gad*
EC 1346	Single	1193	*strA, strB, aadA5, mph(A), sul1, sul2, dfrA17, tet(A), bla*$_{\text{CTX-M-27}}$	Col(BS512), Col156, IncFIA, IncFIB(AP001918)	*cnf1, ireA, iha, gad, vat, senB, sat*
EC 0295	Early onset recurrent	131	*aac(3)-IId, blaTEM-1B, bla*$_{\text{CTX-M-14}}$	IncFIA, IncFIB(AP001918)	*sat, cnf1, iha, iss, gad*
EC 0355	Early onset recurrent	95	*aadA5, aac(3)-IId, sul1, dfrA17, bla*$_{\text{CTX-M-14}}$, *mph(A)*	Col156, IncB/O/K/Z, IncFIB(AP001918), IncFII(29)	*vat, ireA, gad, iss, senB*

EC 0511	Early onset recurrent	131	*strA, strB, aadA5, mph(A), sul1, sul2, dfrA17, tet(A), bla*$_{CTX-M-27}$	Col(KPHS6), IncFIA, IncFIB(AP001918), IncFII(pRSB107)	*sat, iss, gad*
EC 0578	Early onset recurrent	131	*aac(3)-IId, strA, strB, aadA5, mph(A), sul1, sul2, dfrA17, tet(A), bla*$_{CTX-M-14}$, *bla*$_{TEM-1B}$	Col156, IncFIA, IncFIB(AP001918), IncFII(pRSB107)	*nfaE, iha, gad, cnf1, iss, senB, sat*
EC 0632	Early onset recurrent	131	*bla*$_{CTX-M-27}$	Col156, IncFIA, IncFIB(AP001918), IncFII(pRSB107), IncI1-I(Gamma)	*senB, iss, gad*
EC 1132	Early onset recurrent	1193	*mph(A), bla*$^{CTX-M-15}$	Col(BS512), IncFIA, IncFIB(AP001918), IncFII(pHN7A8)	*sat, vat, iha, senB, gad*
EC 1286	Early onset recurrent	131	*aac(3)-IIa, aadA5, mph(A), sul1, dfrA17, tet(A), catB3, aac(6')Ib-cr, bla*$_{OXA-1}$, *bla*$_{CTX-M-15}$	IncFIA, IncFIB(AP001918), IncI1-I(Gamma)	*nfaE, iha, gad, cnf1, iss, senB, sat*
EC 1317	Early onset recurrent	131	*aac(3)-IIa, tet(A), catB3, aac(6')Ib-cr, bla*$_{OXA-1}$, *bla*$_{CTX-M-15}$	IncB/O/K/Z, IncFIA, IncFIB(AP001918)	*sat, cnf1, iha, iss, gad*
EC 1398	Early onset recurrent	131	*strA, strB, aac(3)-IId, aadA5, mph(A), sul1, sul2, dfrA17, tet(A), bla*$_{CTX-M-14}$, *bla*$_{TEM-1B}$	Col156, IncFIA, IncFIB(AP001918), IncFII(pRSB107)	*nfaE, iha, cnf1, gad, iss, senB, sat*
EC 1508	Early onset recurrent	131	*aadA5, aac(3)-IIa, mph(A), sul1, dfrA17, tet(A), catB3, bla*$_{CTX-M-15}$, *bla*$_{OXA-1}$, *aac(6')Ib-cr*	IncFIA, IncFIB(AP001918)	*cnf1, iha, gad, nfaE, iss, senB, sat*
EC 1631	Early onset recurrent	131	*strA, strB, aadA5, mph(A), sul1, sul2, dfrA17, tet(A), bla*$_{CTX-M-27}$	IncFIA, IncFIB(AP001918), IncFII(pRSB107)	*senB, ireA, iha, iss, gad*
EC 0151	Late onset recurrent	131	*aac(3)-IIa, catB3, aac(6')Ib-cr, bla*$_{OXA-1}$, *bla*$_{CTX-M-15}$, *tet(A)*	Col156, IncFIA, IncFIB(AP001918)	*cnf1, iha, nfaE, gad, iss, sat*
EC 1254	Late onset recurrent	648	*aac(3)-IIa, aadA5, mph(A), sul1, dfrA17, tet(B), bla*$_{CTX-M-14}$	Col(MG828), IncFIA, IncFIB(AP001918), IncFII(pRSB107)	*eilA, lpfA, air*
EC 1256	Late onset recurrent	131	*aac(3)-IIa, aadA5, mph(A), sul1, dfrA17, tet(A), catB3, aac(6')Ib-cr, bla*$_{OXA-1}$, *bla*$_{CTX-M-15}$	Col156, ColpVC, IncFIA, IncFIB(AP001918)	*cnf1, iha, gad, iss, senB, sat*
EC 1448	Late onset recurrent	131	*aac(3)-IId, strA, strB, aadA5, mph(A), sul1, sul2, dfrA17, tet(A), bla*$_{CTX-M-3}$, *bla*$_{TEM-1B}$	IncFIB(AP001918), IncFII	*iha*
EC 1563	Late onset recurrent	131	*aac(3)-IIa, aadA5, mph(A), sul1, dfrA17, tet(A), catB3, aac(6')Ib-cr, bla*$_{OXA-1}$, *bla*$_{CTX-M-15}$	Col156, IncFIA, IncFIB(AP001918)	*cnf1, iha, gad, nfaE, iss, senB, sat*

ESBL, extended spectrum β-lactamase; BSI, bloodstream infection; ST, sequence type

Relapses versus reinfections in late onset recurrent BSI

We completed pulsed-field gel electrophoresis (PFGE) evaluations on eight paired isolates of the initial and subsequent episodes from the late onset recurrent BSI group (Figure 2). These evaluations revealed that three pairs (28/28-2, 36/36-2, and 48/48-2) of these isolates produced identical PFGE patterns, ST type, and ESBL genotype, which suggests that these patients were suffering from relapse rather than reinfection.

Figure 2. Dendrogram of XbaI-mediated restriction mapping of the DNA from the *Escherichia coli* specimens isolated from late onset recurrent bloodstream infections (N = 107). Pulsed-field gel electrophoresis was performed using a Lambda Ladder (Promega, Fitchburg, WI, USA). Abbreviations: ST, sequence type; ESBL, Extended spectrum β-lactamase.

Discussion

This study revealed that recurrent *E. coli* BSI was not uncommon in this cohort and that the rate of early onset recurrence was similar to that of late onset recurrence. Our evaluations also identified several independent risk factors for recurrent *E. coli* BSI including the presence of a vascular catheter, ESBL phenotype, and SOFA score. In addition, molecular evaluation of these samples revealed that multidrug-resistant *E. coli* ST131 strains, which produce ESBL, were more common in early onset recurrent BSI than in late onset recurrent BSI or single episode BSI. PFGE analysis suggested that some of the cases in the late onset recurrent BSI group could be attributed to relapse rather than reinfection. WGS revealed that

ESBL–producing *E. coli* strains were characterized by a variety of resistance genes and plasmid types.

Recurrence is a serious complication associated with BSI with the rate of recurrence often being dependent on the causative pathogen, definition of recurrence, and follow-up duration. However, recurrence rates for several common pathogens have already been established, these include *Staphylococcus aureus* (5–12%) [20-22], *Streptococcus pneumonia* (2.3%) [23], *Acinetobacter baumannii* (5.6%) [24], and *Salmonella* species (15.7%) [25]. We found that 6.3% of the patients with *E. coli* BSI evaluated in this study experienced some recurrence of their infection. Our study also defined the recurrence rate for two specific subgroups of these patients, early onset (3.6%) and late onset (3.5%) recurrences. To date, there is limited information describing changes in the recurrence rate in response to time following the first episode, which may be clinically relevant and could help plan an appropriate follow-up for *E. coli* BSI patients.

We demonstrated that ESBL phenotype was an important risk factor for recurrent BSI. Maslow et al. showed that impaired host defense responses were closely associated with recurrent episodes [9]. A study by Sanz-Garcı´a et al. suggested that male sex, the presence of hematological malignancy, inadequate antibiotic use, and extra-urinary infections were all independent risk factors for recurrence [11]. As these studies were conducted before the widespread dissemination of ESBL–producing *E. coli* [26-28], they could not evaluate the association between ESBL phenotype and recurrence. Patients with BSI caused by ESBL–producing *E. coli* may have an increased risk for subsequent recurrent BSI due to the multidrug-resistant traits of these bacteria. There is some evidence to support this hypothesis, as other studies have shown that antibiotic resistance observed in the initial *E. coli* infection increases the risk of recurrent BSI [12,13].

Recurrent BSI can be caused by infection with identical (relapse) or different (reinfection) isolates, usually determined by molecular typing. PFGE analysis was used to evaluate these two types of recurrences in the available late onset samples. Although relapse was less common than reinfection, it was still observed at a substantial rate. Reinfection is associated with a higher degree of severity of illness and may be difficult to modify and/or prevent [6]. In contrast, relapse may occur from the remnant foci of the initial infection which are produced following the undertreatment of the original BSI. This implies that appropriate therapy, such as the elimination of any residual infection foci, may reduce the rate of relapse.

ESBL–producing *E. coli* BSI has increased over the past decade owing to the widespread dissemination of the ST 131 clone containing CTX-M-15 [26-28]. To date, limited data are available for the rate of ESBL–producing *E. coli* in recurrent BSI. We showed that as much as 33.3% of our recurrent cases were the result of ESBL–producing *E. coli* and that CTX-M-15 was the most common ESBL genotype in these patients. Multiple virulence factors may be involved in the survival of ESBL–producing *E. coli* and our data suggests that *gad,* which is associated with the protection of cells in acidic environments, was the most prevalent virulence gene in our samples. We also identified strains positive for *iss*, *iha*, and *sat*, which are involved in immune evasion, adhesion, and protease production, respectively [29,30].

Although our study provides some much-needed insight into recurrent BSI, it does have some limitations. First, not all of the *E .coli* BSI isolates were included in the analysis; therefore, there may have been a selection bias. Second, we could not perform PFGE on the *E. coli* isolates from patients with early onset recurrent BSI. However, we postulated that most of the early onset recurrent BSIs were infected by identical strains. *E. coli* isolated from early onset recurrent BSI was highly resistant to third-generation cephalosporin or ciprofloxacin, which are most commonly used as initial therapy after the identification of

gram-negative BSI. This means that the initial empirical antibiotic therapy was likely ineffective, therefore, it is reasonable to assume that the majority of the early onset recurrence cases might be caused by remnants of their initial infection. Third, we showed that the presence of a vascular catheter was associated with recurrent *E. coli* BSI, which agrees with the findings of previous studies [13,31]. However, the number of patients with a vascular catheter was too low to draw any conclusive statements. Therefore, the findings related to those few patients will need to be interpreted with caution.

Conclusions

Our evaluations identified several independent risk factors for recurrent *E. coli* BSI, which may be helpful to healthcare providers when planning patient follow-up after initial *E. coli* BSI. The detection of variable molecular feature in the *E. coli* strains responsible for these recurrent infections suggests the need for an in-depth study into these strains to identify the microbiological determinants associated with recurrent *E. coli* BSI.

Acknowledgements

None.

Competing interests

The authors have no conflicts of interest to declare.

Funding

This work was supported by the National Health Insurance Service Ilsan Hospital grant (No. NHIMC2019CR012 to YAK). The funder played no role in the study design, data analysis, or

preparation of the manuscript.

Authors' contributions

YAK and YSP, conception and study design; HC, YAK and YHS, data acquisition; YCK and YAK, data analysis and interpretation; YCK, draft preparation; HC, HL and KL, manuscript review and revision; YAK and YSP, final approvals.

Supporting information

S1 Table. Comparison of patients with early- and late-onset recurrent *Escherichia coli* BSIs

Reference

1. Kaper JB, Nataro JP, Mobley HLT. Pathogenic Escherichia coli. Nature Reviews Microbiology. 2004;2: 123-140. doi:10.1038/nrmicro818
2. Bou-Antoun S, Davies J, Guy R, Johnson AP, Sheridan EA, Hope RJ. Descriptive epidemiology of Escherichia coli bacteraemia in England, April 2012 to March 2014. Euro Surveill. 2016;21. doi:10.2807/1560-7917.ES.2016.21.35.30329 PMID:27608263
3. Uslan DZ, Crane SJ, Steckelberg JM, Cockerill FR, 3rd, St Sauver JL, Wilson WR, et al. Age- and sex-associated trends in bloodstream infection: a population-based study in Olmsted County, Minnesota. Arch Intern Med. 2007;167: 834-839. doi:10.1001/archinte.167.8.834 PMID:17452548
4. Berger J, Diab-Elschahawi M, Blacky A, Pernicka E, Spertini V, Assadian O, et al. A matched prospective cohort study on Staphylococcus aureus and Escherichia coli bloodstream infections: extended perspectives beyond resistance. Am J Infect Control. 2010;38: 839-845. doi:10.1016/j.ajic.2010.04.212 PMID:20650546
5. Bonten M, Johnson JR, van den Biggelaar AHJ, Georgalis L, Geurtsen J, de Palacios PI, et al. Epidemiology of Escherichia coli Bacteremia: A Systematic Literature Review. Clin Infect Dis. 2021;72: 1211-1219. doi:10.1093/cid/ciaa210 PMID:32406495
6. Wendt C, Messer SA, Hollis RJ, Pfaller MA, Wenzel RP, Herwaldt LA. Recurrent gram-negative bacteremia: incidence and clinical patterns. Clin Infect Dis. 1999;28: 611-617. doi:10.1086/515152 PMID:10194087
7. Cattaneo C, Antoniazzi F, Tumbarello M, Skert C, Borlenghi E, Schieppati F, et al. Relapsing bloodstream infections during treatment of acute leukemia. Ann Hematol. 2014;93: 785-790. doi:10.1007/s00277-013-1965-0 PMID:24288110

8. Marschall J, Doherty J, Warren DK. The epidemiology of recurrent Gram-negative bacteremia in a tertiary-care hospital. Diagn Microbiol Infect Dis. 2010;66: 456-459. doi:10.1016/j.diagmicrobio.2009.12.005 PMID:20226339

9. Maslow JN, Mulligan ME, Arbeit RD. Recurrent Escherichia coli bacteremia. J Clin Microbiol. 1994;32: 710-714. doi:10.1128/jcm.32.3.710-714.1994 PMID:7910828

10. Samet A, Sledzińska A, Krawczyk B, Hellmann A, Nowicki S, Kur J, et al. Leukemia and risk of recurrent Escherichia coli bacteremia: genotyping implicates E. coli translocation from the colon to the bloodstream. Eur J Clin Microbiol Infect Dis. 2013;32: 1393-1400. doi:10.1007/s10096-013-1886-9 PMID:23649557

11. Sanz-Garcia M, Fernandez-Cruz A, Rodriguez-Creixems M, Cercenado E, Marin M, Munoz P, et al. Recurrent Escherichia coli bloodstream infections: epidemiology and risk factors. Medicine (Baltimore). 2009;88: 77-82. doi:10.1097/MD.0b013e31819dd0cf PMID:19282697

12. Woudt SHS, de Greeff SC, Schoffelen AF, Vlek ALM, Bonten MJM, Infectious Diseases Surveillance Information System-Antimicrobial Resistance Study G. Antibiotic Resistance and the Risk of Recurrent Bacteremia. Clin Infect Dis. 2018;66: 1651-1657. doi:10.1093/cid/cix1076 PMID:29228127

13. Lee CH, Su LH, Chen FJ, Tang YF, Chien CC, Liu JW. Clinical and microbiologic characteristics of adult patients with recurrent bacteraemia caused by extended-spectrum β-lactamase-producing Escherichia coli or Klebsiella pneumoniae. Clin Microbiol Infect. 2015;21: 1105.e1101-1108. doi:10.1016/j.cmi.2015.07.025 PMID:26271718

14. Woudt SHS, de Greeff SC, Schoffelen AF, Vlek ALM, Bonten MJM, Infectious Diseases Surveillance Information System–Antimicrobial Resistance Study G.

Antibiotic Resistance and the Risk of Recurrent Bacteremia. Clinical Infectious Diseases. 2018;66: 1651-1657. doi:10.1093/cid/cix1076

15. Charlson ME, Pompei P, Ales KL, MacKenzie CR. A new method of classifying prognostic comorbidity in longitudinal studies: development and validation. J Chronic Dis. 1987;40: 373-383. doi:10.1016/0021-9681(87)90171-8 PMID:3558716

16. Horan TC, Andrus M, Dudeck MA. CDC/NHSN surveillance definition of health care-associated infection and criteria for specific types of infections in the acute care setting. Am J Infect Control. 2008;36: 309-332. doi:10.1016/j.ajic.2008.03.002 PMID:18538699

17. CLSI. Performance Standards for Antimicrobial Susceptibility Testing. 27th ed. CLSI supplement M100. Wayne, PA: Clinical and Laboratory Standards Institute; 2017.

18. Kim H, Kim YA, Park YS, Choi MH, Lee GI, Lee K. Risk Factors and Molecular Features of Sequence Type (ST) 131 Extended-spectrum beta-Lactamase-producing Escherichia coli in Community-onset Bacteremia. Sci Rep. 2017;7: 14640. doi:10.1038/s41598-017-14621-4 PMID:29116143

19. Johnson JR, Murray AC, Gajewski A, Sullivan M, Snippes P, Kuskowski MA, et al. Isolation and molecular characterization of nalidixic acid-resistant extraintestinal pathogenic Escherichia coli from retail chicken products. Antimicrob Agents Chemother. 2003;47: 2161-2168. doi:10.1128/aac.47.7.2161-2168.2003 PMID:12821463

20. Welsh KJ, Skrobarcek KA, Abbott AN, Lewis CT, Kruzel MC, Lewis EM, et al. Predictors of relapse of methicillin-resistant Staphylococcus aureus bacteremia after treatment with vancomycin. J Clin Microbiol. 2011;49: 3669-3672. doi:10.1128/jcm.05287-11 PMID:21865420

21. Fowler VG, Jr., Kong LK, Corey GR, Gottlieb GS, McClelland RS, Sexton DJ, et al. Recurrent Staphylococcus aureus bacteremia: pulsed-field gel electrophoresis findings in 29 patients. J Infect Dis. 1999;179: 1157-1161. doi:10.1086/314712 PMID:10191218
22. Chang FY, Peacock JE, Jr., Musher DM, Triplett P, MacDonald BB, Mylotte JM, et al. Staphylococcus aureus bacteremia: recurrence and the impact of antibiotic treatment in a prospective multicenter study. Medicine (Baltimore). 2003;82: 333-339. doi:10.1097/01.md.0000091184.93122.09 PMID:14530782
23. King MD, Whitney CG, Parekh F, Farley MM. Recurrent invasive pneumococcal disease: a population-based assessment. Clin Infect Dis. 2003;37: 1029-1036. doi:10.1086/377736 PMID:14523766
24. Lai CC, Hsu HL, Tan CK, Tsai HY, Cheng A, Liu CY, et al. Recurrent bacteremia caused by the Acinetobacter calcoaceticus-Acinetobacter baumannii complex. J Clin Microbiol. 2012;50: 2982-2986. doi:10.1128/jcm.01194-12 PMID:22760035
25. Hsu RB, Chen RJ, Chu SH. Risk factors for recurrent bacteremia in adult patients with nontyphoid salmonellosis. Am J Med Sci. 2004;328: 315-318. doi:10.1016/s0002-9629(15)33940-9 PMID:15599326
26. Thaden JT, Fowler VG, Sexton DJ, Anderson DJ. Increasing Incidence of Extended-Spectrum β-Lactamase-Producing Escherichia coli in Community Hospitals throughout the Southeastern United States. Infect Control Hosp Epidemiol. 2016;37: 49-54. doi:10.1017/ice.2015.239 PMID:26458226
27. Rodríguez-Baño J, Picón E, Gijón P, Hernández JR, Ruíz M, Peña C, et al. Community-onset bacteremia due to extended-spectrum beta-lactamase-producing Escherichia coli: risk factors and prognosis. Clin Infect Dis. 2010;50: 40-48. doi:10.1086/649537

PMID:19995215

28. Colpan A, Johnston B, Porter S, Clabots C, Anway R, Thao L, et al. Escherichia coli sequence type 131 (ST131) subclone H30 as an emergent multidrug-resistant pathogen among US veterans. Clin Infect Dis. 2013;57: 1256-1265. doi:10.1093/cid/cit503 PMID:23926176

29. Grant MA, Weagant SD, Feng P. Glutamate decarboxylase genes as a prescreening marker for detection of pathogenic Escherichia coli groups. Applied and environmental microbiology. 2001;67: 3110-3114. doi:10.1128/AEM.67.7.3110-3114.2001 PMID:11425729

30. Fröding I, Hasan B, Sylvin I, Coorens M, Nauclér P, Giske CG. Extended-Spectrum-β-Lactamase- and Plasmid AmpC-Producing Escherichia coli Causing Community-Onset Bloodstream Infection: Association of Bacterial Clones and Virulence Genes with Septic Shock, Source of Infection, and Recurrence. Antimicrob Agents Chemother. 2020;64. doi:10.1128/aac.02351-19 PMID:32423949

31. Hanna H, Afif C, Alakech B, Boktour M, Tarrand J, Hachem R, et al. Central venous catheter-related bacteremia due to gram-negative bacilli: significance of catheter removal in preventing relapse. Infect Control Hosp Epidemiol. 2004;25: 646-649. doi:10.1086/502455 PMID:15357155

Figure1

Click here to access/download;Figure;Figure 1.jpg

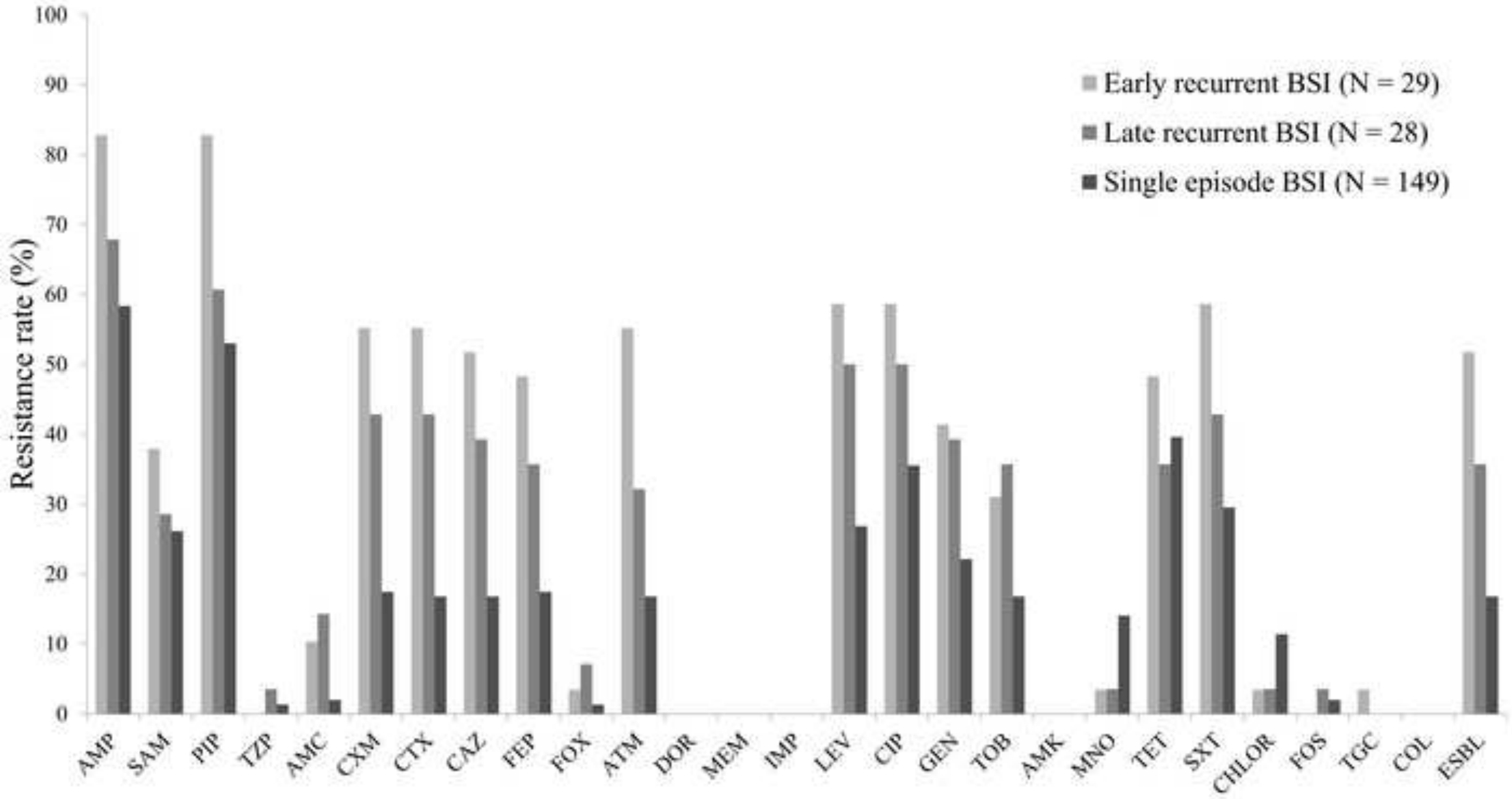

Figure2

Click here to access/download;Figure;Figure 2.jpg

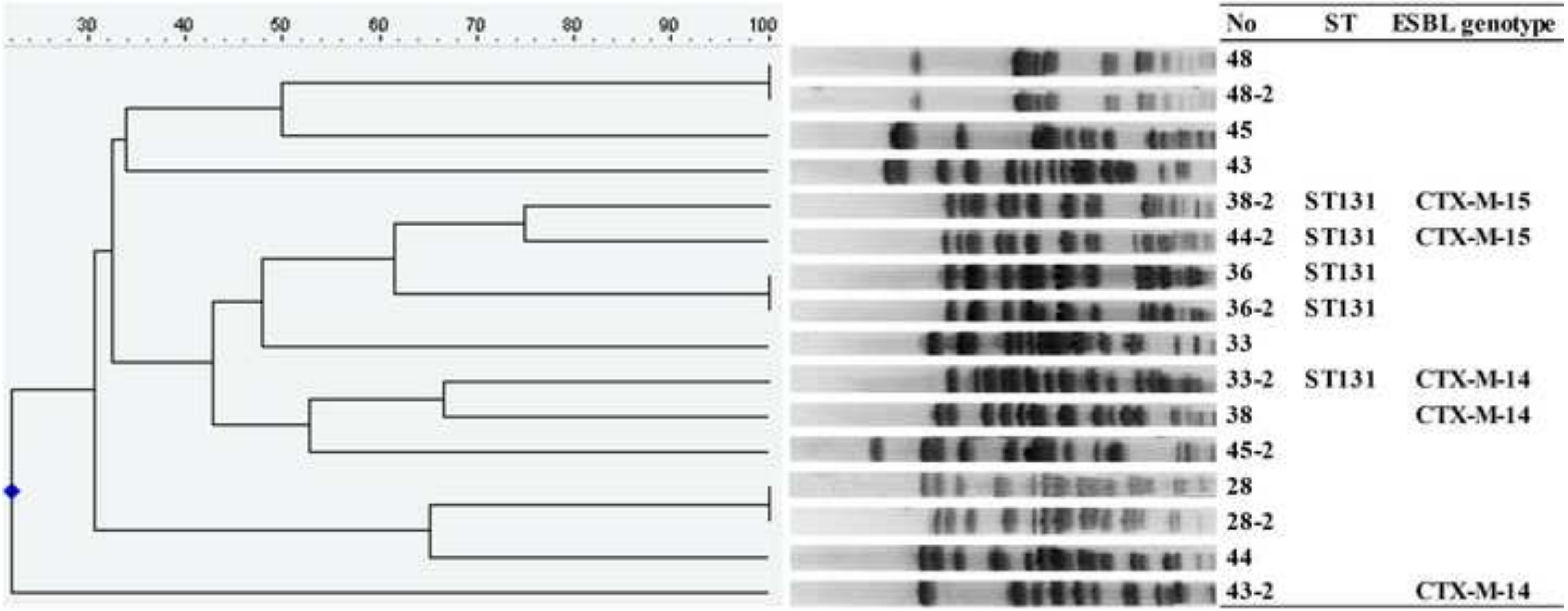

Click here to access/download
Supporting Information
S1 Table.docx
