# Supplementary material for: Risk factors and microbiological features of recurrent Escherichia coli bloodstream infections
Source: PLoS One. 2023 Jan 10;18(1):e0280196. doi: 10.1371/journal.pone.0280196 (PMC9831297; doi:10.1371/journal.pone.0280196)
Supplement: Attachment — Submitted filename: Response to reviewers.docx [file pone.0280196.s005.docx]

**Detailed Response to Reviewers**

**Note:** The reviewers’ comments are in boxes, and our responses are in plain text.

We thank the reviewers for their thoughtful comments and suggestions, which have enhanced the manuscript and produced a balanced account of the research.

**Reviewer #1:**

**Comment #1**

As it is a retrospective study, details of how the follow up data was collected needs to be explained.

**Authors’ response to comment #1>**

To address your comment. The Department of Laboratory Medicine in Ilsan Hospital, where this study was conducted, has been collecting *E. coli* strains that caused bloodstream infection for research. Since they were not specimen of human origin, informed consent was not required from the subjects for collection. Patients’ information related to *E. coli* strains was collected retrospectively through medical records.

**Comment #2**

No where have the authors juxtaposed the different resistance or virulence genes among the groups,to compare. with an appropriate statistical test.

Table 1. just lists the various genes. This may be modified to a comparative table.

**Authors’ response to comment #2>**

Please verify whether your comment is about Table 4 and not Table 1. If you are referring to Table 4, we could not compare antimicrobial resistance genes, plasmid types, and virulence factors detected in each of the ESBL-producing E. coli isolates from each of the different BSI groups due to limited numbers of isolates included in the WGS test. For reference, below is a table that shows various antimicrobial resistance genes detected in the different BSI groups.

|  | Single episodes  (n = 11) | Early onset recurrence (n = 11) | Late onset recurrence (n= 5) |
| --- | --- | --- | --- |
| *aac(3)-IIa* | 6 | 3 | 4 |
| *aac(3)-IId* | 0 | 4 | 1 |
| *aac(6')Ib-cr* | 7 | 3 | 3 |
| *aacA4* | 1 | 0 | 0 |
| *aadA5* | 5 | 7 | 4 |
| *catA2* | 1 | 0 | 0 |
| *catB3* | 7 | 3 | 3 |
| *mph(A)* | 5 | 8 | 4 |
| *sul1* | 5 | 7 | 4 |
| *sul2* | 2 | 4 | 1 |
| *tet(A)* | 5 | 7 | 4 |
| *tet(B)* | 1 | 0 | 1 |
| *strA* | 2 | 4 | 1 |
| *strB* | 2 | 4 | 1 |
| *dfrA14* | 1 | 0 | 0 |
| *dfrA17* | 5 | 7 | 4 |
| *blaTEM-1B* | 0 | 3 | 1 |
| *blaOXA-1* | 7 | 3 | 3 |
| *blaCTX-M-3* | 0 | 0 | 1 |
| *blaCTX-M-14* | 0 | 4 | 1 |
| *blaCTX-M-15* | 7 | 4 | 3 |
| *blaCTX-M-27* | 2 | 3 | 0 |
| *blaCTX-M-55* | 1 | 0 | 0 |

Therefore, we decided to present Table 4 as supplementary data (S2 Table). We further describe this limitation in the “Discussion” section, as follows (Page 17, Line 322–326):

“Third, we could not compare antimicrobial resistance genes, plasmid types, and virulence factors detected in each of the ESBL-producing *E. coli* isolates from each of the different BSI groups due to limited numbers of isolates included in the WGS test. Further studies are needed to provide more information on molecular characteristics of ESBL-producing *E. coli* causing BSI.”

**Comment #3**

Minor points have been highlighted in the attached manuscript.

-There is no shortening in “Short Title”

**Authors’ response to comment #3>**

We agree and we have revised the short title, as follows:

“Recurrent *Escherichia coli* bloodstream infections”

**Comment #4**

Minor points have been highlighted in the attached manuscript.

-Grammar error in “Abstract”

**Authors’ response to comment #4>**

We concur and we have corrected the sentence as follows (Page 3 Line 31–32):

Original: “We also identify the ST131 and ESBL genotype to…”

🡪 Revised: “We also identified the ST131 and ESBL genotype to…”

**Comment #5**

Minor points have been highlighted in the attached manuscript.

-What statistical test was applied to note significant differences in Table 3?

**Authors’ response to comment #5>**

We acknowledge your detailed comment. To address your comment, we performed a statistical test, results of which are show below:

|  | Single episode  BSI  (N = 149) | Early onset recurrent  BSI  (N = 29) | Late onset recurrent BSI  (N = 28) | *P* |
| --- | --- | --- | --- | --- |
| ST131, n (%) | 34 (22.8) | 12 (41.4) | 7 (25) | 0.112 |
| O25 | 29 (19.5) | 12 (41.4) | 6 ( 21.4) | 0.036 |
| O16 | 5 (3.4) | 0 | 1 (3.6) | 0.832 |
| ESBL genotype, n (%) | 25 (16.8) | 12 (41.4) | 7 (25) | 0.011 |
| CTX-M-3 | 0 | 0 | 1 (3.6) | 0.136 |
| CTX-M-14 | 5 (3.4) | 3 (10.3) | 3 (10.7) | 0.078 |
| CTX-M-15 | 17 (11.4) | 5 (17.2) | 3 (10.7) | 0.617 |
| CTX-M-27 | 2 (1.3) | 4 (13.8) | 0 | 0.012 |
| CTX-M-55 | 1 (0.7) | 0 | 0 | >0.999 |

We also modified the manuscript according to the statistical test result (Page 12, Line 210–215).

Original: “ST131 strains were more frequently observed in the early onset group (N = 12, 41.4%) when compared to the late onset recurrent episode (N = 7, 25.0%) and single episode (N = 34, 22.8%) groups. *E. coli* with an ESBL genotype were also more commonly observed in the early onset recurrent episode (N = 12, 41.4%) group when compared with either the late onset recurrent episode (N = 7, 25%) or single episode BSI (N = 25, 16.8%) groups.”

🡪 Revised: ‘The ST131–025 strains were more frequently observed in the early onset group (N = 12, 41.4%) when compared to the late onset recurrent episode (N = 6, 21.4%) and single episode (N = 29, 19.5%) groups (*P* = 0.036). *E. coli* with an ESBL genotype were also more commonly observed in the early onset recurrent episode (N = 12, 41.4%) group when compared with either the late onset recurrent episode (N = 7, 25%) or single episode BSI (N = 25, 16.8%) groups (*P* = 0.011).”
