# Supplementary material for: Risk factors and microbiological features of recurrent Escherichia coli bloodstream infections
Source: PLoS One. 2023 Jan 10;18(1):e0280196. doi: 10.1371/journal.pone.0280196 (PMC9831297; doi:10.1371/journal.pone.0280196)
Supplement: Attachment — Submitted filename: PLOS-Risk factors and microbiological features.docx [file pone.0280196.s006.docx]

1. E. coli strains that caused bloodstream infection for research. Since they were not specimen of human origin, informed consent was not required from the subjects for collection🡪 How did this reflect BS infections in specific patients ?

However the following stamen is contradictory: Our study cohort consisted of adult patients (age ≥18 92 years) who presented at the Ilsan Hospital with positive blood cultures for E. coli at least 93 once over the course of a two-year period.

Was any permission sought and granted from the hospital authorities to use patient data? If so this must be mentioned.

The paper still lacks in clarity with regards to the genetic studies on resistance and recurrence outcome . This may be highlighted .
