# Supplementary material for: Risk factors and microbiological features of recurrent Escherichia coli bloodstream infections
Source: PLoS One. 2023 Jan 10;18(1):e0280196. doi: 10.1371/journal.pone.0280196 (PMC9831297; doi:10.1371/journal.pone.0280196)
Supplement: Attachment — Submitted filename: Response to Reviewers.docx [file pone.0280196.s007.docx]

**Detailed Response to Editor and Reviewer**

**Note:** The reviewer’s comments are in boxes, and our responses are in plain text.

We thank the editor and reviewers for their thoughtful comments and suggestions, which have enhanced the manuscript and produced a balanced account of the research.

**Journal Requirements:**

Please review your reference list to ensure that it is complete and correct. If you have cited papers that have been retracted, please include the rationale for doing so in the manuscript text, or remove these references and replace them with relevant current references. Any changes to the reference list should be mentioned in the rebuttal letter that accompanies your revised manuscript. If you need to cite a retracted article, indicate the article’s retracted status in the References list and also include a citation and full reference for the retraction notice.

**Authors’ response>**

We have reviewed and changed the reference list. We found that reference numbers 12 and 14 were duplicated, so we corrected the reference number after deleting one of them. These changes were also applied to the introduction, methods, results, and discussion.

**Additional Editor Comments:**

Please clarify regarding patient consent and add a note on the limitation of the study.

**Authors’ response>**

In response to the reviewer’s comments, statements regarding patient consent and descriptions of the limitation of this study are listed below.

**Reviewer #1:**

**Comment #1**

E. coli strains that caused bloodstream infection for research. Since they were not specimen of human origin, informed consent was not required from the subjects for collection How did this reflect BS infections in specific patients ?

However the following stamen is contradictory: Our study cohort consisted of adult patients (age ≥18 92 years) who presented at the Ilsan Hospital with positive blood cultures for E. coli at least 93 once over the course of a two-year period.

Was any permission sought and granted from the hospital authorities to use patient data? If so this must be mentioned.

**Authors’ response to comment #1>**

We regret to inform you that there were errors and omissions in our prior explanation.

*E. coli* strains identified in blood culture were of human origin. However, since bacteria isolated from the human body do not contain the genetic information of humans, there are no restrictions on the collection or use of the collected strains. This matter is addressed in detail in the bylaws of the Institutional Review Board (by which this study was approved). Young Ah Kim (Department of Laboratory Medicine at Ilsan Hospital) harvested several strains isolated from blood culture, including *E. coli* for another study. *E. coli* strains analyzed for this study were already collected and matched with the ID number of patients, who had experienced bloodstream infections (BSIs) due to corresponding strains. Therefore, clinical information on patients with BSIs caused by a specific strain could likewise be obtained through a medical record review. The aforementioned information was disclosed when receiving the Institutional Review Board approval for this study.

**Comment #2**

The paper still lacks in clarity with regards to the genetic studies on resistance and recurrence outcome. This may be highlighted.

**Authors’ response to comment #2>**

We attempted to evaluate the genetic characteristics of ESBL-producing *E. coli* strains from each of the different BSI groups and determine the effects of genetic factors on recurrence. However, we could not determine the effect of antimicrobial resistance genes, plasmid types, and virulence factors detected in ESBL-producing *E. coli* isolates on recurrence due to the limited number of isolates included in the WGS test.

We further describe this limitation in the “Discussion” section of the paper (Page 17, Line 322–326):

“Third, we could not determine the effect of antimicrobial resistance genes, plasmid types, and virulence factors detected in ESBL-producing E. coli isolates on recurrent BSI due to limited numbers of isolates included in the WGS test. This limitation precluded comprehensive and accurate explanation for genetic characteristics of E. coli causing recurrent BSI.”
